# Early relapse is an adverse prognostic marker in systemic immunoglobulin light chain (AL) Amyloidosis

**DOI:** 10.1038/s41375-021-01497-7

**Published:** 2022-01-06

**Authors:** Sriram Ravichandran, Steven Law, Shameem Mahmood, Brenden Wisniowski, Darren Foard, Marianna Fontana, Ana Martinez-Naharro, Carol Whelan, Julian D. Gillmore, Helen J. Lachmann, Philip N. Hawkins, Ashutosh D. Wechalekar

**Affiliations:** National Amyloidosis Centre, University College London (Royal Free Campus), London, UK

**Keywords:** Epidemiology, Myeloma

## To the Editor:

Systemic Immunoglobulin light chain amyloidosis (AL) is a protein-misfolding disorder associated with an underlying monoclonal B-cell or plasma cell dyscrasia. Whilst the prognosis of AL has markedly improved with novel agents [1], it remains incurable with a relapsing-remitting course, necessitating multiple treatment lines. Baseline cardiac bio-markers [2] and the depth of response to initial therapy [2–4] are critical prognostic variables. Early relapse confers a poor prognosis in Myeloma [5–7]. There is little information on the impact of response durability on outcomes in AL. The dual pathology in AL (the clonal biology and the organ dysfunction due to amyloid deposition) lead to a complex interplay of factors. We have recently demonstrated that outcomes after each relapse episode are similar, i.e., deaths occur at each relapse [8]. Here, we assess the impact of timing of relapse on outcomes in a cohort of AL patients treated with upfront Bortezomib.

## Patients & methods

All patients treated with frontline Bortezomib in 2010–2019 at the National Amyloidosis Centre, UK, are included in the analysis. Patients with primary refractory disease, those with ≤24 months follow up and continuing response and those who received 2nd line therapy for reasons other than progression are excluded from the analysis. We report response (complete response- CR; very good partial response- VGPR; partial response-PR) based on the validated ISA criteria. [2] We report haematologic progression based on the consensus criteria published by the ISA (ISA Criteria, Table SA1) [9]. We also report progression based on the recently published “high-risk” dFLC (difference between involved and uninvolved light chains) criteria (Pavia Criteria, Table SA1) [10]. We define progression as earlier of the two criteria (conventional or high-risk dFLC). We analysed the survival of patients based on the cut-offs reported in Myeloma (≤12 months & ≤24 months). Due to the high and ongoing early mortality from end-organ damage in cardiac AL (even in patients in a deep response as we and other authors have previously reported), which persists at 12 months from diagnosis [8, 11], to keep the focus on the impact of clonal progression in this manuscript and to avoid introducing a bias due to the organ related mortality independent of progression, we chose 24 months (Early relapse, ER) as the optimal cut-off for this analysis. Late relapse (LR) was defined as patients who either had haematologic progression after 24 months or had not relapsed beyond 24 months to the last follow up. A detailed description of the patients and methods is available in the Supplementary Appendix.

## Results & discussion

In total, 560 patients are analysed here (Fig. SA1). In total, 513/560 (91.6%) patients were treated with CyBorD. The other regimens were—Bortezomib-Dexamethasone (4.5%), Bortezomib-Thalidomide-Dexamethasone (2.1%), Bortezomib-Melphalan-Prednisolone (0.7%), Bortezomib-Adriamycin-Dexamethasone (0.5%), Bortezomib-Rituximab-Dexamethasone (0.4%) and Bortezomib-Lenalidomide-Dexamethasone (0.2%).

331/560 (59.1%) patients fulfilled one of the two progression criteria (ISA or Pavia criteria). 267 patients fulfilled the ISA progression criteria, and 235 patients fulfilled the Pavia criteria (Figs. SA2 & 3). 171/331 (51.7%) patients had progressed by both criteria at the time of this analysis. 64/331 (19.3%) had only progressed by the Pavia criteria, and 96/331 (29%) had only progressed by the ISA criteria. Of the 171 patients who progressed by both criteria, 73/171 (42.7%) fulfilled both criteria at the same time, 44/171 (25.7%) patients progressed by the ISA criteria before the Pavia criteria (median difference between the two progression criteria was 4 months, range 1–28 months) and 54/171 (31.6%) progressed by the Pavia criteria before the ISA criteria (median difference 3.5 months, range 1–22 months).

250 (44.6%) and 310 (55.4%) patients had early (ER) and late (LR) relapse, respectively. In the LR group, 81 (26.1%) patients had progressed, and 229 (73.9%) patients were in continuing response. 38/331 (11.5%) patients had died after progression without receiving further therapy (Table SA2 and Fig. SA4). 40/560 (7.1%) patients received a stem cell transplant. The baseline characteristics of the two groups are captured in Table 1 (significant differences in bold). The ER group had more advanced cardiac disease (*p* < 0.005), a higher serum M-protein (*p* = 0.023) and higher dFLC (*p* < 0.005). The median bone marrow plasma cell percentage was not significantly different in either group; but, the ER group had a higher proportion of patients with plasma cells > 20% (33.9% vs. 23.5%, *p* = 0.02). The ER group also had a lower proportion of patients with deep haematologic response after 1st line therapy (*p* < 0.005).

**Table 1:** Baseline characteristics.

	Full cohort (*n* = 560)			OR for early relapse OR (95% CI)			
Characteristic *n* (%)/(range)	Early relapse (*n* = 250)	Late relapse (*n* = 310)	*p* value	Univariate analysis	*p* value	Multivariate analysis	*p* value
Age, y	66 (29-84)	66 (36-88)	NS	1.007 (0.989–1.024)	0.450		
Gender							
Male	151 (60.4)	162 (52.3)	NS	1.393 (0.994–1.953)	0.054		
Female	99 (39.6)	148 (47.7)		Reference category			
Performance status							
ECOG 0-2	238 (95.2)	301 (97.1)	NS	Reference category			
ECOG > 2	12 (4.8)	9 (2.9)		1.686 (0.699–4.069)	0.245		
Cardiac involvement	149 (59.6)	164 (52.9)	NS	1.313 (0.938–1.840)	0.113		
Mayo stage (European modification)							
I	39 (12.6)	74 (23.9)	0.01	0.589 (0.383–0.906)	0.016		
II	102 (40.8)	116 (37.4)	NS	1.153 (0.819–1.622)	0.415		
IIIa	81 (32.4)	99 (31.9)	NS	1.022 (0.715–1.459)	0.907		
IIIb	28 (11.2)	21 (6.8)	NS	1.736 (0.960–3.138)	0.068		
*Revised Mayo stage*							
I	31 (12.4)	82 (26.5)	<0.005	0.394 (0.250–0.619)	<0.005		
II	76 (30.4)	94 (30.3)	NS	1.004 (0.699–1.442)	0.984		
III	76 (30.4)	84 (27.1)	NS	1.175 (0.813–1.698)	0.390		
IV	67 (26.8)	50 (16.1)	<0.005	1.904 (1.261–2.875)	0.002		
NT-proBNP, ng/L	1353 (12–37290)	474 (4–93602)	<0.005	1.884 (1.512–2.348)	<0.005		
High-sensitivity cardiac troponin T, ng/L	47 (3–742)	38 (1–689)	0.012	1.554 (1.096–2.202)	0.013		
Renal involvement	172 (68.8)	225 (72.6)	NS	0.833 (0.578–1.201)	0.328		
Serum creatinine, µmol/L	96 (40–1124)	96 (27–610)	NS	1.000 (0.998–1.002)	0.992		
Proteinuria, g/24 h	2.8 (0–29.8)	3.8 (0.1–36)	NS	0.988 (0.954–1.023)	0.484		
Liver involvement	24 (9.6)	47 (15.2)	0.049	0.594 (0.352–1.002)	0.051		
Alkaline Phosphatase, U/L	82 (41–1035)	88 (16–1178)	NS	0.999 (0.997–1.000)	0.060		
GI Involvement	11 (4.4)	8 (2.6)	NS	1.737 (0.688–4.388)	0.243		
Autonomic nervous system involvement	19 (7.6)	16 (5.2)	NS	1.511 (0.760–3.004)	0.239		
Peripheral nervous system involvement	19 (7.6)	17 (5.5)	NS	1.418 (0.721–2.789)	0.312		
Soft tissue involvement	46 (18.4)	40 (12.9)	NS	1.522 (0.960–2.414)	0.074		
Heavy chain isotype							
IgA	42 (16.8)	45 (14.5)	NS	1.189 (0.752–1.880)	0.459		
IgD	1 (0.4)	1 (0.3)	NS	1.241 (0.077–19.940)	0.879		
IgG	87 (34.8)	106 (34.2)	NS	1.027 (0.724–1.458)	0.881		
IgM	4 (1.6)	8 (2.6)	NS	0.614 (0.183–2.063)	0.430		
LC	57 (22.8)	67 (21.6)	NS	1.071 (0.718–1.599)	0.737		
None	59 (23.6)	83 (26.8)	NS	0.845 (0.575–1.242)	0.391		
Serum monoclonal protein, g/L	9 (IF-45)	6.5 (IF-38)	0.023	2.110 (1.104–4.032)	0.024	2.636 (1.033–6.732)	0.012
Light chain isotype							
Kappa	56 (22.4)	60 (19.4)	NS	Reference category			
Lambda	194 (77.6)	250 (80.6)		0.831 (0.552–1.252)	0.377		
dFLC, mg/L	205 (1–13007)	128.8 (1–5316)	<0.005	1.938 (1.458–2.578)	<0.005		
dFLC > 180 mg/l	138 (55.2)	121 (39)	<0.005	1.925 (1.373–2.698)	<0.005		
dFLC > 500 mg/l	66 (26.4)	49 (15.8)	0.002	1.911 (1.262–2.893)	0.002		
dFLC > 1000 mg/l	31 (12.4)	16 (5.2)	0.002	2.601 (1.388–4.875)	0.003		
FLC ratio ≥ 100	33 (13.2)	20 (6.5)	0.006	2.205 (1.231–3.949)	0.008		
Bone marrow plasma cells at diagnosis (morphology)	*n* = 174 10 (1–90)	*n* = 226 10 (1–95)	NS	1.644 (0.985–2.745)	0.057		
Bone marrow plasma cells >10%	116 (66.7)	130 (57.5)	NS	1.477 (0.979–2.228)	0.063		
Bone marrow plasma cells > 20%	59 (33.9)	53 (23.5)	0.02	1.675 (1.079–2.599)	0.021		
Number of cycles of chemotherapy (1st line)	6 (2-9)	6 (2-11)	NS				
≥VGPR after 1^st^ line	153 (61.2)	270 (87.1)	<0.005	0.234 (0.154–0.355)	<0.005		
dFLC < 10 mg/l after 1st line	59 (23.6)	214 (69)	<0.005	0.139 (0.095–0.202)	<0.005	0.122 (0.063–0.235)	<0.005
iFLC < 20 mg/l after 1st line	40 (16)	153 (49.4)	<0.005	0.195 (0.130–0.293)	<0.005		

*dFLC* the difference between involved and uninvolved light chains, *ECOG* Eastern co-operative oncology group, *NT-proBNP* N-terminal pro-brain natriuretic peptide, *iFLC* involved free light chain, *NS* not significant

**Fig. 1:**
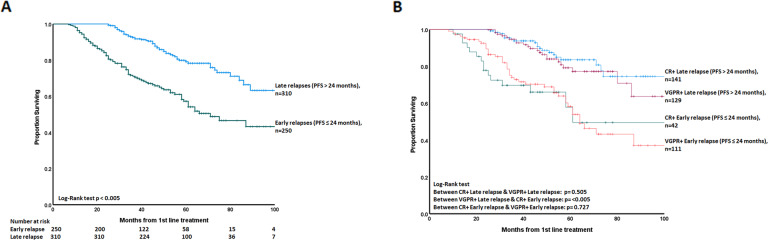
Impact of early relapse on overall survival. **A** Kaplan–Meier curve showing the impact of early (≤24 months) vs late (>24 months) relapse on OS from 1st line. Patients with early relapse had a significantly poorer survival than the late relapses—median OS 71 months (95% CI 53.53–88.46 months) vs not reached (*p* < 0.005). 100%, 100% and 80% of LR patients were alive at the end of 1, 2 & 5 years, respectively. **B** Kaplan–Meier curve showing the impact of early (≤24 months) vs late (>24 months) relapse on OS from 1st line, stratified by the haematologic response (CR & VGPR) after 1st line. Patients who relapsed early had a significantly poorer survival than those who relapsed late, irrespective of their initial haematologic response (*p* < 0.005). There was no significant difference in survival between CR+ early response & VGPR+ early response- median OS 61 months vs 64 months (95% CI 53. 91–74.08 months), *p* = 0.727. Similarly, there was no significant difference in survival between CR + late response and VGPR + late response- median OS 109 months vs not reached, *p* = 0.505. VGPR + late response had a superior survival than CR + early response- median OS not reached vs 61 months, *p* < 0.005. Of, 100%, 100% and 80% of VGPR + late response were alive at the end of 1, 2 & 5 years, respectively.

The median OS of the entire cohort was 109 months. Patients with ER had significantly poorer survival than the LR patients- median OS 71 months vs not reached after 1st line (*p* < 0.005) (Fig. 1A). ER patients had poorer survival irrespective of the depth of response to 1st line therapy (*p* < 0.005). ER patients had poorer survival than LR patients after 2nd line also (Fig. SA5). The outcomes of patients achieving a CR or VGPR were not significantly different within ER or LR groups, but patients with CR/VGPR in the LR group had significantly better survival than patients with CR in the ER group—median OS not reached vs 61 months (*p* < 0.005) (Fig. 1B and SA6).

In a univariate model (Table 1), disease stage [Mayo stage I (*p* = 0.016), revised Mayo stage I (*p* < 0.005), & revised Mayo stage IV (*p* = 0.002)], NT-proBNP (*p* < 0.005), Troponin T (*p* = 0.013), serum M-protein (*p* = 0.024), FLC ratio ≥100 (*p* = 0.008), dFLC (*p* < 0.005), bone marrow plasma cells > 20% (*p* = 0.021), ≥ VGPR after 1st line (*p* < 0.005), dFLC < 10 mg/l (*p* < 0.005), and iFLC <20 mg/l (*p* < 0.005) were significant predictors of early relapse. In a Multivariate model including NT-proBNP, Troponin T, baseline dFLC, serum M-protein and bone marrow plasma cells >20%, serum M-protein [OR 2.460 (95% CI 1.076-5.624), *p* = 0.033] & NT-proBNP [OR 1.506 (95% CI 1.036-2.188), (*p* = 0.032)] were significant predictors of early relapse. When haematologic response (dFLC <10 mg/l or iFLC <20 mg/L (separate models for each respectively)) was added to the above model (instead of the baseline dFLC), we found serum M-protein [OR 2.636 (95% CI 1.033-6.732), *p* = 0.043] & dFLC <10 mg/l [OR 0.122 (95% CI 0.063-0.235), (*p* < 0.005)] (as well as iFLC <20 mg/L (OR 0.242, 95% CI 0.126–0.465, *p* < 0.005)) were significant predictors of early relapse.

These data show that AL patients with haematologic relapse within 24 months of initial treatment have significantly poorer survival -this appears to be linked to the biology of the underlying clone as the early relapsing patients have higher presenting dFLC, serum M-protein and bone marrow plasma cells. They also have worse organ involvement with higher NT-proBNP at presentation. The clonal biology and the degree of organ involvement are likely related since patients with bone marrow plasma cells >20% have a greater cardiac involvement [12]. The depth of response to the initial treatment is a critical determinant of response durability- the ER group had a poorer depth of response to initial chemotherapy, and a deep response (dFLC <10 mg/L) was independently predictive of LR. Patients with advanced cardiac involvement are often sicker; inevitably have dose modifications/treatment delays in addition to having a higher clonal burden- both likely to impact haematologic response. The patients in this cohort were treated at their local hospitals, and we do not have access to the dose intensity; therefore, we cannot test the above hypothesis. We acknowledge this limitation of the present data. The present data are congruent with the published data in Myeloma [5–7, 13].

Whilst there are no published reports on response durability impacting outcomes in AL to compare with the current analysis, the Mayo clinic group has reported that patients with higher presenting plasma cell percentages have poorer outcomes [12] and higher cardiac involvement, consistent with the current study. The presence of cytogenetic abnormalities (hyperdiploidy or t(11;14)) is associated with poorer outcomes in AL [14]. The lack of cytogenetic data (at presentation and relapse) in the current cohort is a significant limitation.

Since the current cohort was exclusively treated with bortezomib upfront, we cannot comment on the impact of other therapies on ER/LR. As clonal burden at presentation appears to be a marker of early progression, treatment strategies may need modulating on initial clonal markers (in addition to organ involvement that has dominated therapeutic adjustments). As deeper responses translated to LR, Daratumumab-CyBorD, which is highly effective in achieving deep responses, is an attractive option; but, longer follow up is needed for progression data in the Andromeda study [15]. Other options may include deferred autologous stem cell transplantation (SCT) consolidation in those not eligible for an upfront SCT. There is little data on maintenance therapy to delay relapse, and prospective trials are required.

The study’s retrospective nature, absence of cytogenetic data at diagnosis and clonal evolution at progression remain limitations of this analysis.

In conclusion, response durability is an equally important variable (along with the depth of response) in assessing prognosis in AL. Regimens capable of inducing a deep response are available and should be adopted early. The early relapses should be considered for clinical trials that can identify treatments with the potential to overcome the high-risk biology of the disease.

## Supplementary information

Supplementary Appendix
