# Supplementary material for: Early relapse is an adverse prognostic marker in systemic immunoglobulin light chain (AL) Amyloidosis
Source: Leukemia. 2022 Jan 6;36(4):1180–4. doi: 10.1038/s41375-021-01497-7 (PMC8979816; doi:10.1038/s41375-021-01497-7)
Supplement: Supplementary file 1 — Supplementary Appendix [file 41375_2021_1497_MOESM1_ESM.docx]

**Supplementary Appendix**

**Early relapse is an adverse prognostic marker in systemic immunoglobulin light chain (AL) Amyloidosis**

**Table of contents**

Patients and methods Page 2

Results Page 5

Table SA1 Page 7

Table SA2 Page 7

Figure Legends Page 8

Figure SA1 Page 10

Figure SA2 Page 10

Figure SA3 Page 11

Figure SA4 Page 11

Figure SA5 Page 12

Figure SA6 Page 12

References Page 13

**Patients and Methods**

The National Amyloidosis Centre (NAC), UK is a referral centre for newly diagnosed AL patients in the United Kingdom. Patients diagnosed with AL amyloidosis are seen at diagnosis and then every six months for a comprehensive assessment. The ALchemy study is an ongoing prospective observational study of newly diagnosed AL amyloidosis seen at the UK National Amyloidosis Centre (NAC) from April 2009. The study is approved by the relevant institutional review board and all patients provided written informed consent per the declaration of Helsinki.

The AL diagnosis was confirmed at the NAC by biopsy immunohistochemistry. Proteomics was performed where required. All patients underwent an assessment of organ involvement/function at baseline- liver & renal function, 24-hour urine collection for proteinuria, cardiac biomarkers, echocardiography (and cardiac MRI where required) and SAP scan. Serological assessment of the plasma cell dyscrasia was also performed at baseline- serum & urine protein electrophoresis & immunofixation and serum free light chains. All these investigations were performed at NAC where the data is stored and analysed. Bone marrow examination was not performed at the NAC. However, all patients had a bone marrow examination at baseline at their local hospital and the results were sent for the NAC for inclusion in the ALchemy database. As the plasma cell burden was small in majority of cases, cytogenetics was not routinely performed by the local labs. All patients underwent a similar assessment of organ function and serological markers of the plasma cell dyscrasia at each follow up visit. Also, serum samples from patients were received prior to each cycle of chemotherapy for assessment of serum free light chains and monoclonal protein in serum.

Organ involvement and responses were defined by ICC. ^1,2^ The European modification of Mayo 2004 staging was used with Stage III stratified into IIIa (NT-proBNP < 8500ng/L) and IIIb (NT-proBNP ≥ 8500ng/L).^3^ We report the responses based on the validated criteria reported by Palladini et al.^4,5^ Organ responses are reported based on the criteria published by Palladini et al. ^4^ and Gertz et al. ^2^

Patients were treated at their local centres as per nationally agreed protocols. Patients were treated with intravenous Bortezomib until 2013, after which time subcutaneous Bortezomib was used. All decisions for any change in treatment were made with input from the multidisciplinary team at the UK National Amyloidosis Centre and considered the patients’ haematologic response (or progression), degree of organ damage, whether there was an improvement in organ function and functional status. However, dose modifications of chemotherapy agents, steroid doses and the number of treatment cycles were at the discretion of the locally treating physician.

We also collected data on response assessment, survival status, and dates of death/last known follow up and subsequent treatments.

Overall survival (OS) was calculated from the date of 1^st^ line therapy to death or last follow up. Patients who were alive at their last follow up are censored.

All statistical analysis was performed using SPSS version 27 (IBM Inc, USA). As NT-proBNP, Troponin T, and dFLC levels were not normally distributed, we log-transformed these values for analysis. The method of Kaplan & Meier was used to generate survival curves. The two-sided log-rank test was used to assess statistical significance between survivals. All reported p values are two-sided with the conventional significance of ≤ 0.05. The independent t-test was used to test for a significant difference in means between the subjects. The Chi-Square tests was used the check for a significant difference in categorical variables between the subjects. We used regression models (binary logistic regression) to analyse the factors predicting early relapse.

**Results**

The median age of the entire cohort was 66 years (29-88 years). 313 (55.9%) and 397 (70.9%) patients had cardiac and renal involvement. 49 (8.8%) patients had advanced cardiac involvement (Mayo IIIb). The median NT-proBNP, Troponin T (TNT), proteinuria, creatinine, serum monoclonal protein, and difference between involved and uninvolved light chains (dFLC) were 771 ng/l (4-93602 ng/l), 43 ng/l (1-742 ng/l), 3.5 gm/24h (0-36 gm/24h), 96 µmol/l (27-1124 µmol/l), 8 gm/l (1-45 gm/l), and 157.3 mg/l (1-13007 mg/l), respectively. The baseline bone marrow burden was available in 400 (71.4%) patients. The median bone marrow plasma cells were 10% (1-95%).

All patients received frontline treatment with Bortezomib based regimens based on nationally agreed protocols. The doses of steroids was at the discretion of the individual physicians. The number of cycles were also at the discretion of the individual physicians. Patients received a median of 6 cycles (range 2-12 cycles).

183 (32.7%), 240 (42.9%) & 137 (24.5%) patients had achieved a CR, VGPR and PR after 1^st^ line. 273 (48.8%) and 193 (34.5%) patients had dFLC < 10 mg/L and iFLC < 20 mg/L, respectively. 230 (41.1%) patients received 2^nd^ line treatment.

**2^nd^ line treatment**

331 patients fulfilled either of the progression criteria, 229 patients were in a continuing response at the time of this analysis (Consort diagram SA4). 230/331 (69.5%) patients received 2^nd^ line treatment following haematologic progression. 38/101 (37.6%) patients who did not receive 2^nd^ line treatment following progression had died after a median of 7.5 months from the time of progression (range 0-65 months). 182/250 (72.8%) patients in the early relapse group received 2^nd^ line treatment following progression. Of the 68 patients in the early relapse group who did not receive 2^nd^ line treatment, 30/68 (44.1%) patients had died after a median of 7 months from progression (range 0-65 months).

Table SA2 shows the treatments received in 2^nd^ line. Immunomodulatory agents (42.6%) were the commonest treatments received in the 2^nd^ line.

At the start of 2^nd^ line, the median NT-proBNP, Creatinine, Proteinuria and dFLC were 1538 ng/l (42-117874 ng/l), 102.5 µmol/l (41-945 µmol/l), 1.4 g/24h (0-21.9 gm/24h), and 89 mg/l (4.6-6064 mg/l). 100/221 (45.2%) patients had evidence of organ progression at the start of 2^nd^ line therapy. Data on organ progression was unavailable for 9 patients (3.9%). There was no difference between the ER and LR in respect of organ progression at 2^nd^ line: 82/176 (46.6%) patients with early relapse had organ progression at 2^nd^ line, compared to 18/45 (40%) patients with late relapse (p=0.427).

ER patients had significantly poorer survival than LR patients after 2^nd^ line therapy- median OS not reached in both groups (p=0.031) (Figure SA5).

**Table SA1 : Definition of haematologic progression- ISA criteria^1^ and Pavia criteria^6^**

| **ISA criteria** | **Pavia criteria** |
| --- | --- |
| 1. From CR, any detectable monoclonal protein or abnormal free light chain ratio (light chain must double) 2. From PR, 50% increase in serum M protein to >0.5 g/dl or 50% increase in urine M protein to >200 mg/day (a visible peak must be present) 3. Free light chain increase of 50% to >100 mg/l | Need to fulfil all three of the following:-   1. dFLC > 20 mg/l (absolute value) 2. dFLC > 20% of baseline dFLC 3. dFLC > 50% of nadir value reached at best response |

**Table SA2: 2nd line treatments**

| Agent | n (%) |
| --- | --- |
| Proteasome inhibitor | 22 (9.6) |
| Immunomodulatory agents | 98 (42.6) |
| Autologous stem cell transplant | 21 (9.1) |
| Daratumumab | 41 (17.8) |
| Alkylator | 48 (20.9) |

**Figure Legends**

**Figure SA1:** Consort diagram showing the analysable cohort. 1276 patients received frontline Bortezomib in the period 2010-2019. 381 (27.7%) patients had primary refractory disease, 243 (19%) patients with a continuing response had a follow up < 24 months and 92 (7.2) patients received 2^nd^ line treatment for reasons other than progression. These three groups were excluded from the analysis, leaving an analysable cohort of 560 patients. 250 (44.6%) and 310 (55.4%) patients had ER and LR, respectively.

**Figure SA2:** Kaplan-Meier curve showing the impact of early (≤ 24 months) vs late (> 24 months) relapse (Pavia criteria) on OS from 1^st^ line. Patients with early relapse had a significantly poorer survival than the late relapses - median OS 64 months (95% CI 51.42-76.57 months) vs 109 months (95% CI 58.41-159.58 months), p = 0.001.

**Figure SA3:** Kaplan-Meier curve showing the impact of early (≤ 24 months) vs late (> 24 months) relapse (ISA criteria) on OS from 1^st^ line. Patients with early relapse had a significantly poorer survival than the late relapses - median OS 71 months (95% CI 49.89-92.10 months) vs 109 months (95% CI 68.41-149.58 months), p < 0.005.

**Figure SA4:** Consort diagram showing the patients who received 2^nd^ line treatment.

**Figure SA5:** Kaplan-Meier curve showing the impact of early (≤ 24 months) vs late (> 24 months) relapse on OS from 2^nd^ line. Patients with early relapse had a significantly poorer survival from 2^nd^ line therapy- median OS not reached in both groups (p=0.031) (Figure 1B). 84%, 70% & 51% of early relapses were alive at the end of 1, 2 & 5 years, compared to 98%,98%, & 70% of late relapses, respectively.

**Figure SA6:** Kaplan-Meier curve showing the impact of early (≤ 24 months) vs late (> 24 months) relapse on OS from 1^st^ line in patients with PR after 1^st^ line therapy. There was no significant difference in survival between the early and late relapses- median OS 74 months vs 84 months (95% CI 75.97-92.02) (p = 0.292).

**References**

1. Comenzo RL, Reece D, Palladini G, et al. Consensus guidelines for the conduct and reporting of clinical trials in systemic light-chain amyloidosis. *Leukemia*. 2012;26(11):2317-2325.

2. Gertz MA, Comenzo R, Falk RH, et al. Definition of organ involvement and treatment response in immunoglobulin light chain amyloidosis (AL): A consensus opinion from the 10th International Symposium on Amyloid and Amyloidosis. *American Journal of Hematology*. 2005;79(4):319-328.

3. Wechalekar AD, Schonland SO, Kastritis E, et al. A European collaborative study of treatment outcomes in 346 patients with cardiac stage III AL amyloidosis. *Blood*. 2013;121(17):3420-3427.

4. Palladini G, Dispenzieri A, Gertz MA, et al. New criteria for response to treatment in immunoglobulin light chain amyloidosis based on free light chain measurement and cardiac biomarkers: impact on survival outcomes. *J Clin Oncol*. 2012;30(36):4541-4549.

5. Palladini G, Schönland SO, Sanchorawala V, et al. Clarification on the definition of complete haematologic response in light-chain (AL) amyloidosis. *Amyloid*. 2021:1-2.

6. Palladini G, Milani P, Foli A, et al. Presentation and outcome with second-line treatment in AL amyloidosis previously sensitive to nontransplant therapies. *Blood*. 2018;131(5):525-532.
